# Interaction between Curcumin and β-Casein: Multi-Spectroscopic and Molecular Dynamics Simulation Methods

**DOI:** 10.3390/molecules26165092

**Published:** 2021-08-22

**Authors:** Ruichen Zhao, Xiaoli Qin, Jinfeng Zhong

**Affiliations:** 1 College of Food Science, Southwest University, Chongqing 400715, China; zrchen99@foxmail.com (R.Z.); qinxl@swu.edu.cn (X.Q.); 2 National Demonstration Center for Experimental Food Science and Technology Education, Southwest University, Chongqing 400715, China

**Keywords:** hydrophobic interaction, fluorescence quenching, molecular docking, secondary structure content, thermodynamic parameters

## Abstract

Effect of temperature and pH on the interaction of curcumin with β-casein was explored by fluorescence spectroscopy, ultraviolet-visible spectroscopy and molecular dynamics simulation. The spectroscopic results showed that curcumin could bind to β-casein to form a complex which was driven mainly by electrostatic interaction. The intrinsic fluorescence of β-casein was quenched by curcumin through static quenching mechanism. The binding constants of curcumin to β-casein were 6.48 × 10^4^ L/mol (298 K), 6.17 × 10^4^ L/mol (305 K) and 5.73 × 10^4^ L/mol (312 K) at pH 2.0, which was greater than that (3.98 × 10^4^ L/mol at 298 K, 3.90 × 10^4^ L/mol at 305 K and 3.41 × 10^4^ L/mol at 312 K) at pH 7.4. Molecular docking study showed that binding energy of β-casein-curcumin complex at pH 2.0 (−7.53 kcal/mol) was lower than that at pH 7.4 (−7.01 kcal/mol). The molecular dynamics simulation study showed that the binding energy (−131.07 kJ/mol) of β-casein-curcumin complex was relatively low at pH 2.0 and 298 K. α-Helix content in β-casein was decreased and random coil content was increased in the presence of curcumin. These results can promote a deep understanding of interaction between curcumin and β-casein and provide a reference for improving the bioavailability of curcumin.

## 1. Introduction

Curcumin is a natural polyphenol compound derived from turmeric, and commonly used as a natural colorant [1]. Compared with artificial synthetic food additives, curcumin is nontoxic [2] and has anti-inflammatory [3], antibacterial [4], anti-cancer [5], and antioxidant [6] effects. However, curcumin has poor water solubility, low bioavailability and tends to degrade at neutral and basic conditions, limiting its use in functional foods. For example, intestinal epithelial cells can only absorb soluble curcumin, and insoluble curcumin will be excreted in feces. Therefore, the water solubility of curcumin needs to be increased to improve its absorption in the gut [7]. Studies have found that protein can act as an affinity binding agent for polyphenols and may be an ideal carrier for curcumin and other small molecules. Michele et al. [8] added powdered curcumin to buttermilk and increased the bioavailability of curcumin 15 times. Another study found that encapsulating curcumin in β-casein could increase the solubility of curcumin by 2500 times, and the binding of these two compounds increased the antioxidant effects of curcumin [9]. These studies showed that it was feasible to use protein to improve the solubility and bioavailability of curcumin. However, the interaction between curcumin and protein is unclear.

The binding of curcumin to protein can be studied by fluorescence spectroscopy, ultraviolet−visible spectroscopy, circular dichroism and in silico approaches. Molecular dynamics (MD) simulation is a powerful in silico method that uses computers to simulate protein structure and dynamics modeling, which provides detailed information on protein conformational changes and fluctuations [10]. It helps us to understand related experimental phenomena and mechanism. Rajabi et al. [11] found that the conformation of trypsin was changed and unstable when curcumin bound to the it by MD simulation. At the same time, they found that curcumin and trypsin formed a complex mainly driven by hydrogen bond and van der Waals interaction by spectral experiment and MD simulation. Zhang et al. [10] found that hydrophobic interaction and hydrogen bond promoted the myosin-curcumin complex by MD simulation, and they found that the quenching mechanism of curcumin on myosin was static quenching by spectral experiment, which was beneficial to the formation of myosin−curcumin complex. Wang et al. [12] found that there was face-to-face π-π stacking between the tryptophan of nucleocapsid proteins and the aromatic rings of curcumin.

However, little information is available about the influence of pH and temperature on the stability of casein-curcumin complex. The protein structure can be affected by environmental stresses such as temperature and pH [13]. In turn, changes in the protein structure may be related to changes of the stability between the protein and the curcumin, which may alter the performances of curcumin in delivery systems. Therefore, it is important to understand the effect of temperature and pH on the interaction between curcumin and protein.

Previous studies have shown that human serum albumin [14], bovine serum albumin [15], β-lactoglobulin [16], and soy protein isolate [17] could be used as carriers for curcumin. Casein is one of the main proteins in milk [18] and has high nutritional value, good sensory properties, and lower cost. The structural characteristics of casein give it many functional properties, including the combination with ions and small molecules, good surface activity, stability, and gel forming properties. These properties, combined with its high nutritional value, make casein highly sought after in the food industry. For example, Zhang et al. [19] found that exopolysaccharide and casein formed a complex mainly driven by intermolecular hydrogen bond, hydrophobic and electrostatic contacts by infrared spectroscopy. Qin et al. [20] found that caffeic acid and caffeic acid phenethyl ester bound to micellar casein via hydrophobic interactions, and the presence of complexes was confirmed by X-ray diffraction and Fourier transform infrared spectroscopy. However, few reports are available on the interactions between casein and curcumin.

In this paper, spectroscopy was used to study the interactions between curcumin and casein under different temperatures and pH conditions. Furthermore, molecular docking and MD simulation were used to further examine the interactions between β-casein and curcumin at the molecular scale, which was expected to provide a theoretical basis for the widespread use of casein and curcumin complexes in the food industry.

## 2. Results

### 2.1. Fluorescence Spectra

**Figure 1:**
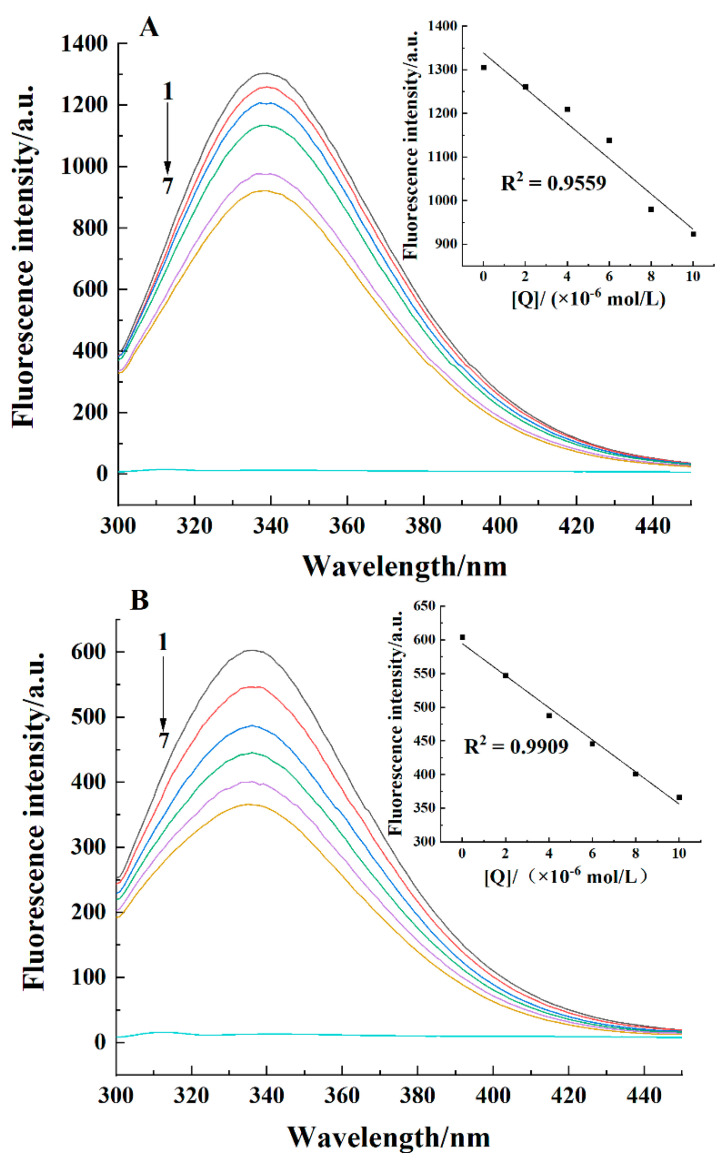
Fluorescence spectra of casein in the absence and presence of curcumin at 298 K at pH 7.4 (**A**) and pH 2.0 (**B**). The curcumin concentrations in curves 1–6 were 0 × 10^−6^, 2 × 10^−6^, 4 × 10^−6^, 6 × 10^−6^, 8 × 10^−6^, and 10 × 10^−6^ mol/L. Curve 7 was for 0.05 mol/L Tris-HCl buffer. Casein concentration was 0.2 g/L.

The endogenous fluorescence of casein comes from tryptophan (Trp), tyrosine (Tyr), and phenylalanine (Phe). The fluorescence information of these amino acids is usually used to study the conformational changes of proteins caused by ligands [21]. Figure 1 shows the effects of curcumin concentration on the fluorescence spectra of casein at 298 K. The fluorescence peak of casein appeared around 337 nm at pH 7.4. In contrast, a fluorescence peak appeared around 334 nm at pH 2.0. As curcumin concentration increased, the fluorescence intensity gradually decreased, indicating an interaction between casein and curcumin.

The effect of pH and temperature on the binding and thermodynamic parameters of casein-curcumin complex is listed in Table 1. *K_sv_* at pH 2.0 was approximately 1.5 times as high as that at pH 7.4, indicating that the complex was more stable at pH 2.0. In addition, *K_sv_* values at both pH values were decreased with increasing temperature, indicating that the complex was more stable at low temperature. The *K_q_* values under the two pH conditions were greater than the maximum diffusion collision quenching constant (2.0 × 10^10^ L/(mol·s)) [22], demonstrating that the quenching mechanism of casein by curcumin was static quenching [23]. The magnitude of *K_b_* was in the range of 10^4^, demonstrating that the binding of curcumin to casein was strong. Moreover, *K_b_* was relatively large at pH 2.0, indicating that curcumin had a relatively great ability to bind to casein at pH 2.0. *K_b_* decreased as temperature increased, showing that low temperature favored the binding of curcumin to casein. The number of binding sites was approximately 1, showing that curcumin and casein could form a complex at a molar ratio of 1:1.

Thermodynamic parameters can be used to judge the main interaction involved in the interaction between curcumin and casein. Δ*H* < 0 and Δ*S* > 0 demonstrated that the binding of curcumin to casein was mainly driven by electrostatic interaction [24]. Δ*H* < 0 indicated that the reaction was exothermic, which also corresponded to the reduction of the binding constant with the increase of temperature. Also, Δ*G* < 0 showed that this binding was spontaneous.

### 2.2. Ultraviolet-Visible Spectra

**Figure 2:**
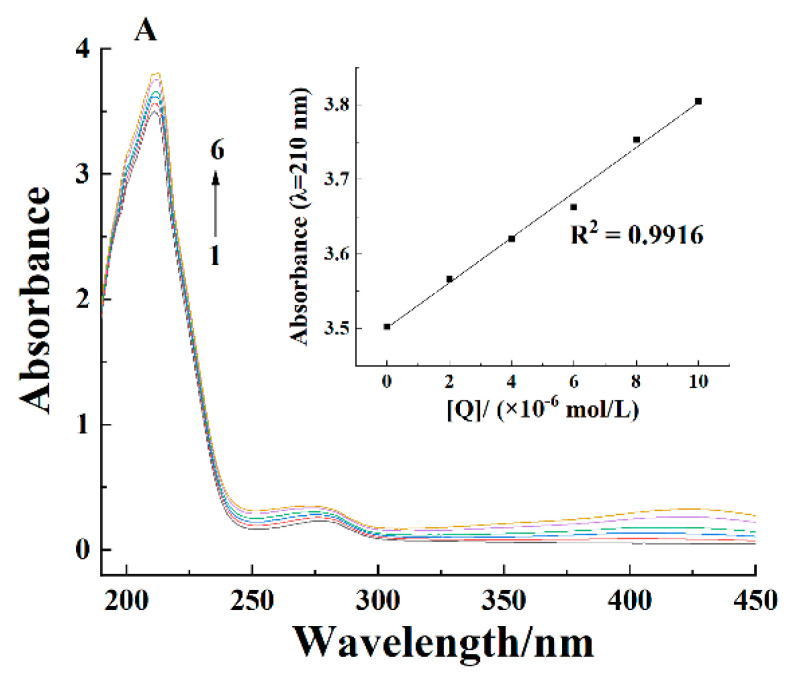

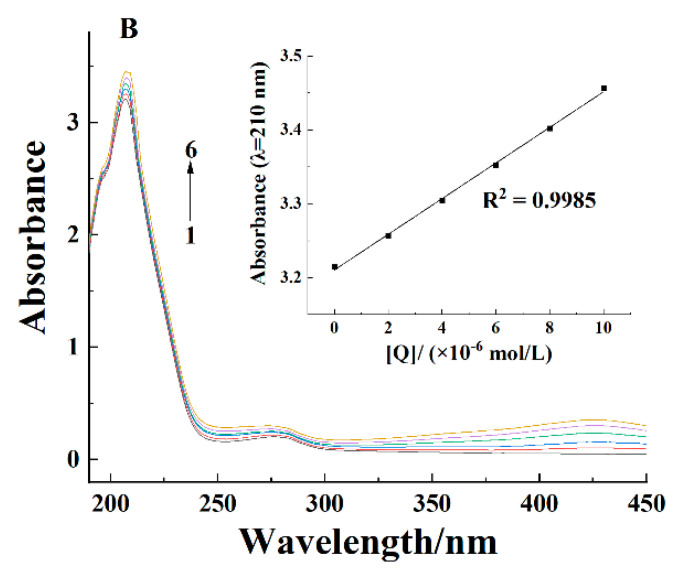
Ultraviolet-visible spectra of casein in the absence and presence of curcumin at 298 K at pH 7.4 (**A**) and pH 2.0 (**B**). The curcumin concentrations in curves 1–6 were 0 × 10^−6^, 2 × 10^−6^, 4 × 10^−6^, 6 × 10^−6^, 8 × 10^−6^, and 10 × 10^−6^ mol/L. Casein concentration was 0.2 g/L.

The ultraviolet-visible spectroscopy is used to get the information about the structural change of proteins [25]. The chromophores of proteins (the aromatic amino acids)—particularly tryptophan—displayed absorption peak around 280 nm [26]. In addition, the α-helical structure also had a characteristic absorption peak around 210 nm, which could roughly determine the binding position of curcumin. Figure 2 shows the effect of curcumin concentration on the ultraviolet-visible spectra of casein. Two absorption peaks could be seen at 210 and 280 nm. The absorbance value increased as curcumin concentration increased, indicating that there were interactions between curcumin and casein, but the peak position remained the same at all curcumin concentrations. This was similar to the result of Cao et al. [21] who studied the effect of eriocitrin concentration on the ultraviolet-visible spectra of β-casein. Significant changes in the spectra occurred before and after curcumin was added, confirming that the quenching mechanism of casein fluorescence by curcumin was static quenching.

### 2.3. Homology Modeling and Evaluation of Three-Dimensional Structure of β-Casein

**Figure 3:**
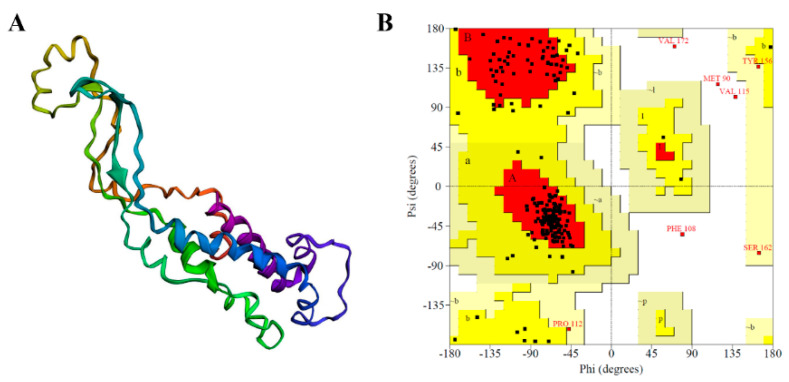
β-casein model (**A**) and its Ramachandran plot (**B**).

Casein is a complex of four kinds of proteins, including α_s1_-casein, α_s2_-casein, β-casein, and κ-casein. β-Casein accounts for more than 25% of the total protein content in milk. Therefore, β-casein is chosen for modeling and subsequent analysis. The PROCHECK program is used to assess the model obtained (Figure 3A) and to generate its Ramachandran plot (Figure 3B). As shown in Figure 3B, black (or red) blocks represent residues; red regions represent optimal regions; bright yellow regions represent rational regions; pale-yellow reasons represent rational regions, and white regions are prohibited regions. 81.6% amino acids were in the optimal region, 15.1% amino acids were in the additional allowed regions, 1.1% amino acids were in the generously allowed regions, and only 2.2% amino acids were in the disallowed regions, which showed that the skeletal construction of the β-casein was reliable.

### 2.4. Molecular Docking

Molecular docking can be used to study the binding site of ligand and receptor. Table 2 shows the binding energy (*E_b_*) and dissociation constant (*K_d_*) obtained from docking results. The greater *K_d_*, the higher was the associated *E_b_*, but the relationship (*E_b_* vs. *K_d_*) was nonlinear, emerging as a monotonically increasing trend. *E_b_* at pH 2.0 (−7.53 kcal/mol) was lower than that at pH 7.4 (−7.01 kcal/mol), indicating that the binding of curcumin to β-casein at pH 2.0 was relatively strong. These results agreed with those obtained from the fluorescence spectroscopy experiment (Table 1), in which *K_b_* at pH 2.0 was greater than that at pH 7.4.

**Figure 4:**
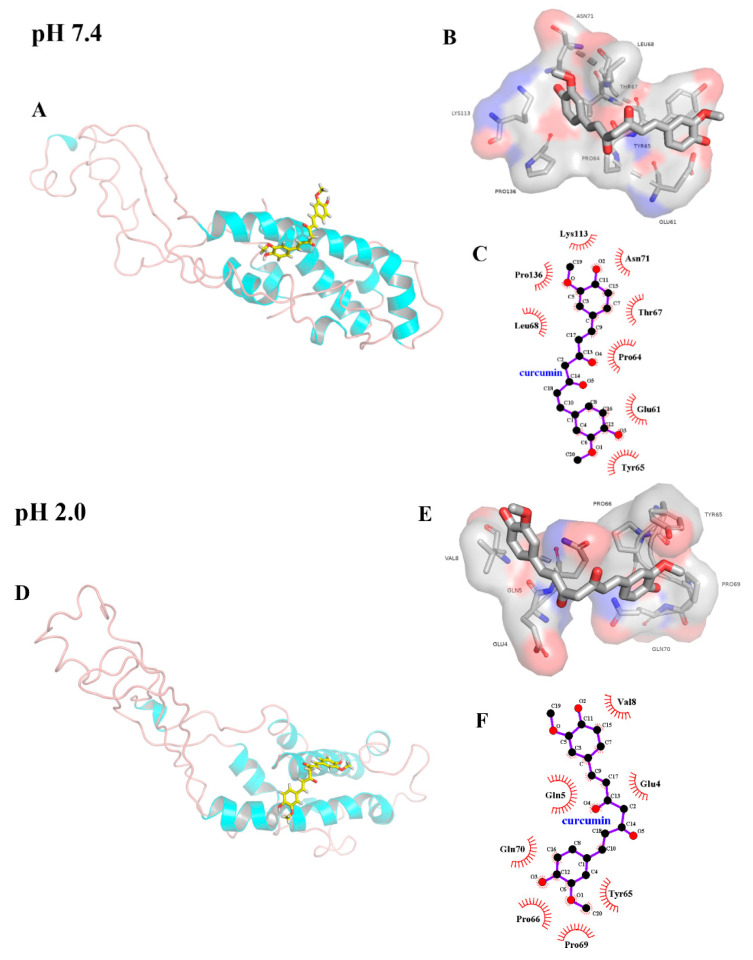
3D image of the best binding pose for curcumin docked into the β-casein at pH 7.4 (**A**) and pH 2.0 (**D**), 3D characterization of the interacted residues between curcumin and β-casein at pH 7.4 (**B**) and pH 2.0 (**E**), and 2D characterization of the interacted residues between curcumin and β-casein at pH 7.4 (**C**) and pH 2.0 (**F**).

Figure 4A–E show the visualization results of the conformation with the lowest energy that are obtained from docking of curcumin to β-casein. Figure 4C,F shows the type of interaction between various amino acids residues and curcumin (only short-distance interactions were shown). At pH 7.4, there were hydrophobic interactions between curcumin and Glu 61, Pro 64, Tyr 65, Thr 67, Leu 68, Asn 71, Lys 113, and Pro 136. At pH 2.0, there were hydrophobic interactions between curcumin and Glu 4, Gln 5, Val 8, Tyr 65, Pro 66, Pro 69, and Gln 70. However, other existing interactions could not be excluded due to the complexity of β-casein structure and the limitations of LigPlot software.

### 2.5. MD Simulation

MD simulation can be used to study the dynamics properties and stability of the docked complex of curcumin and β-casein.

#### 2.5.1. Analysis of the RMSD Value

**Figure 5:**
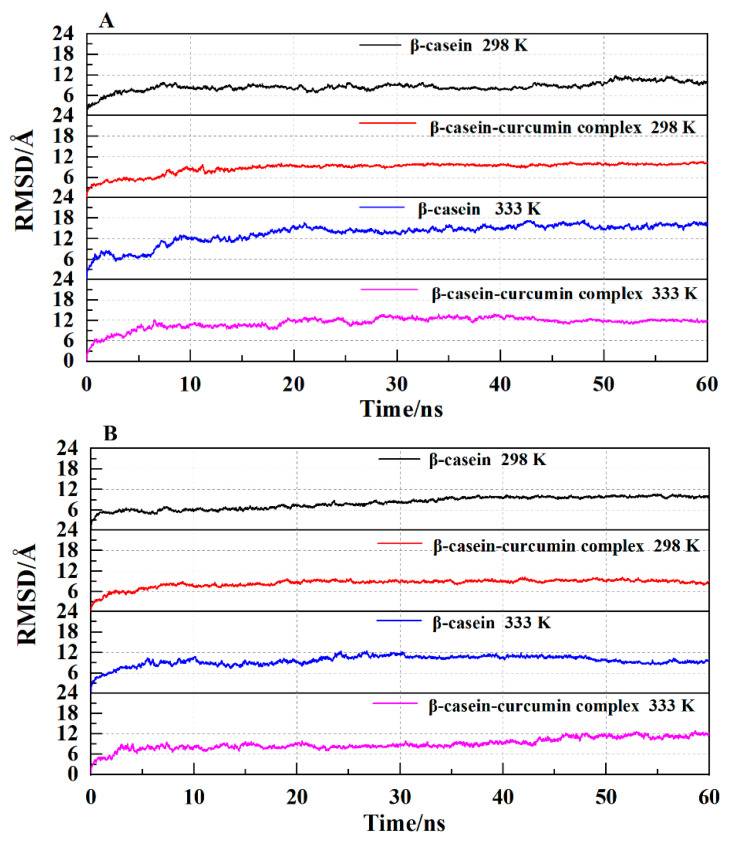
The backbone RMSD of β-casein in the absence/presence of curcumin at pH 7.4 (**A**) and pH 2.0 (**B**).

The trajectory stability is checked by the analysis of the RMSD value as functions of time for β-casein and its complexes with curcumin. Figure 5 shows the changes in the backbone RMSD values of β-casein with time under different pH values. At 298 and 333 K, the final RMSD values of β-casein at pH 7.4 were stable at around 11 Å (after 50 ns) and 17 Å (after 48 ns), whereas the values for the β-casein-curcumin complex were stable at around 9 Å (after 17 ns) and 12 Å (after 30 ns). At pH 2.0, β-casein was stable at around 10.5 Å (after 34 ns) and 9 Å (after 50 ns), while the β-casein-curcumin complex was stable at around 8.5 Å (after 7 ns) and 11.5 Å (after 45 ns). It could be seen that the RMSD of the complex was lower than that of β-casein under the same condition (except at pH 2.0 and 333 K), which suggested that the structure of β-casein was more stable in the presence of curcumin. As temperature increased, the fluctuation of RMSD increased, and the final stable RMSD value also increased. On the other hand, the fluctuation of RMSD of β-casein-curcumin complex at pH 2.0 was significantly lower than that at pH 7.4, indicating that the structure of complex was relatively stable at pH 2.0. The fluctuation of RMSD of the complex before stabilization might be caused by curcumin entering the hydrophobic space of β-casein. During this process, the system changed from an ordered state to a disorderly state, the structure also became looser, being consistent with Δ*S* > 0 obtained from the fluorescence quenching experiment (Table 1). In summary, the RMSD curve of the complex reached a relatively stable value with low fluctuation at pH 2.0 and 298 K, suggesting that the complex was the most stable at this condition.

#### 2.5.2. Hydrogen Bond Analysis

**Figure 6:**
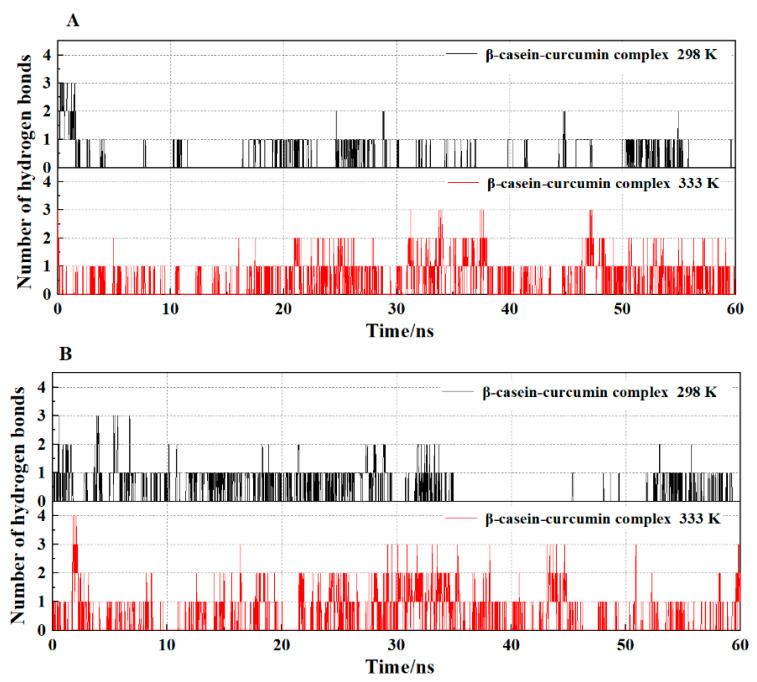
The effects of temperature on the number of hydrogen bonds of β-casein-curcumin complex during 60 ns MD simulation at pH 7.4 (**A**) and pH 2.0 (**B**).

Figure 6 shows the changes in the number of hydrogen bonds in the simulation system with time. The system had a maximum of four hydrogen bonds at pH 2.0 and 333 K, but 0 or 1 hydrogen bond occurred in the system most of the time. Moments where hydrogen bonds had been disappeared were hypothesized to be driven by other interactions. At the same pH, the frequency of hydrogen bonds occurrence significantly increased in the system as temperature increased. At the same temperature, the number of hydrogen bonds and the occurrence frequencies of hydrogen bonds in the system increased at pH 2.0 compared with pH 7.4.

#### 2.5.3. System Energy Analysis

Table 3 shows a summary of various energy items in the system during the last 15 ns MD simulation. Δ*E*, Δ*E_vdw_*, Δ*E_elec_* and Δ*E_inter_* represented the total energy of the system, the energy of van der Waals force, electrostatic energy and internal energy, respectively. Δ*E* was the sum of Δ*E_vdw_*, Δ*E_elec_* and Δ*E_inter_*. The contribution of Δ*E_vdw_* and Δ*E_inter_* to Δ*E* was relatively small, and their positive values were not conducive to the stability of the system. Δ*E_elec_* accounted for a large proportion of the total energy and was a negative value, which was conducive to the stability of the system. It showed that the electrostatic interaction dominated the binding of curcumin to β-casein. This was consistent with the result obtained in Table 1 that the binding of curcumin to casein was mainly driven by electrostatic interaction.

*E_bind_* represents the binding energy after the system reached equilibrium between curcumin and casein. Table 3 shows the average values of binding energy calculated over the last 15 ns MD simulated stable trajectory. At the same temperature, *E_bind_* at pH 2.0 was lower than that at pH 7.4, indicating that the complex was more stable at pH 2.0, which was also consistent with those obtained from the spectroscopic experiment (*K_sv_* and *K_b_* values at pH 2.0 were all greater than those at pH 7.4 in Table 1). As the temperature increased, the binding energy increased. The complex was more stable at pH 2.0 and 298 K than at other conditions.

#### 2.5.4. Analysis of Secondary Structure Content

As shown in Table 4, the presence of curcumin resulted in relatively large changes in the contents of α-helix and coil of β-casein compared with β-sheet and turn contents. The complex showed a decrease in α-helix content and an increase in random coil content compared to β-casein, and the greatest change in secondary structure content was observed at pH 2.0 and 298 K. As temperature increased, α-helix content in the complex increased by 3.66% at pH 7.4 and 4.49% at pH 2.0, respectively. The α-helix content of the complex increased by 4.13% at 298 K and 4.96% at 333 K when pH changed from 7.4 to 2.0. These results suggested that the secondary structure of β-casein was affected by temperature and pH. Combined with the results of hydrogen bond analysis, it was speculated that an increase in temperature or a decrease in pH would promote the formation of hydrogen bonds in the chain (Figure 6), thereby increasing α-helix content.

### 2.6. The Independent Gradient Model Analysis

**Figure 7:**
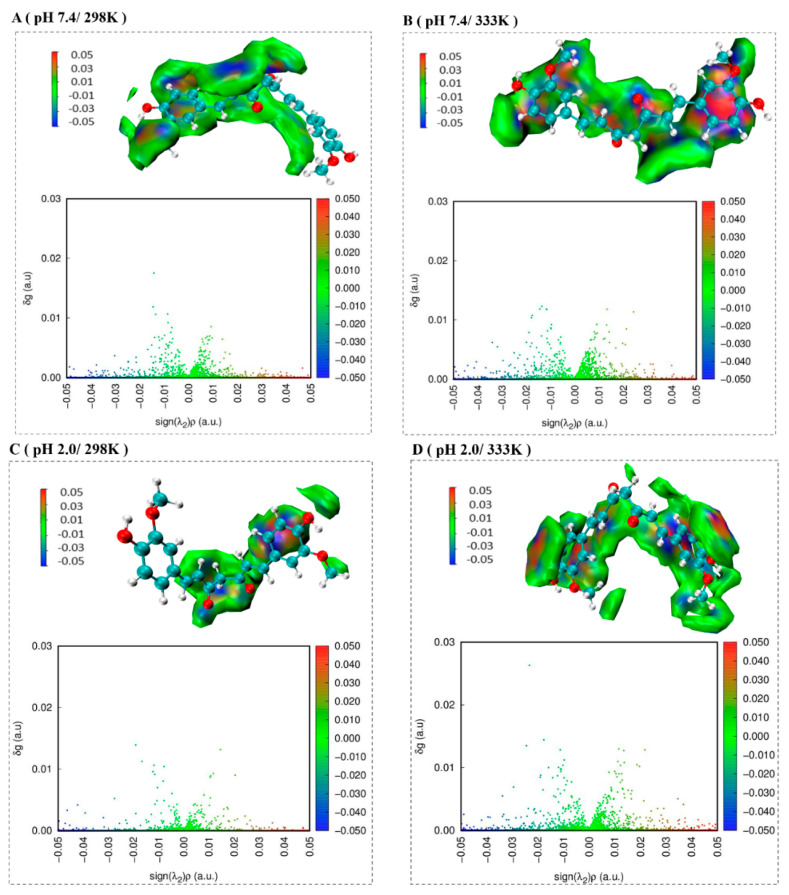
Gradient isosurface and scatter plot of the complex under different pH values and temperatures.

Figure 7 shows the gradient isosurface and scatter plot of the complex. In the gradient isosurface plot, the green region shows van der Waals force, which accounts for most of the area. The red region shows repulsion effect, which is mainly located near the benzene ring. The blue region shows hydrogen bond, which is mainly located near carbonyl oxygen, methyl oxygen, and phenolic hydroxyl oxygen. In the scatter plot, *ρ* stands for the total electron density, λ_2_ stands for the sign of the second eigenvalue of the electron density hessian [27], δ_g_ is the local descriptor which reflects the interaction region between two (or more) fragments [28]. The green, blue and red represented van der Waals force, hydrogen bond and repulsion effect, respectively. The large peak observed in the range of −0.02 ≤ sign(λ_2_)*ρ* ≤ 0.02, indicating that there were many van der Waals interaction in the complex. Some points presented at sign(λ_2_)*ρ* in the range of −0.05 to −0.02 demonstrated the presence of hydrogen bond, which was beneficial to stabilize the complex. However, sign(λ_2_)*ρ* from 0.02 to 0.05 represented the presence of repulsion effect, which was not beneficial to stabilize the complex. In addition, at the same pH, the number of points in sign(λ_2_)*ρ* ranging from −0.05 to −0.02 increased as temperature increased. This suggested that the number of hydrogen bonds increased, which was consistent with changed in the number of hydrogen bonds in MD simulation (Figure 6).

## 3. Materials and Methods

### 3.1. Materials

Casein (CAS#: 9000-71-9) was purchased from Hefei Bomei Biotechnology Co., Ltd., Room 901, Office B-Xinghua International Plaza, No. 50, Hetang Road, Luyang District, Hefei, China. Curcumin (CAS#: 458-37-7, purity ≥ 95%) was obtained from Sinopharm Chemical Reagent Co., Ltd., No. 52, Ningbo Road, Shanghai, China. Absolute ethanol (CAS#: 64-17-5) and hydrochloric acid (CAS#: 7647-01-0) were purchased from Chongqing Chuandong Chemical (Group) Co., Ltd., No. 70, Danzishi New Street, Danzishi Street, Nan’an District, Chongqing, China. Tris (CAS#: 77-86-1) was supplied from Saiguo Biotech Co., Ltd., No. 1, Eighth Alley, Zhangmu Mountain, Cen Village, Guangzhou, Tianhe District, Guangzhou, China. All reagents used in the experiments were analytical grade, and ultrapure water was used for the experiments.

### 3.2. Methods

#### 3.2.1. Solution Preparation

0.2 g of casein powder was dissolved in 0.05 mol/L Tris-HCl buffer with pH of 7.4 and 2.0. The solvent was filled up to 100 mL after the casein had fully dissolved to obtain a 2 g/L casein solution. 0.0368 g curcumin was dissolved in anhydrous ethanol, and the solvent filled up to 1000 mL to obtain a 1.0 × 10^−4^ mol/L curcumin solution.

#### 3.2.2. Fluorescence Spectroscopy

The fluorescence spectra were recorded by a fluorescence spectrophotometer (F-2500, Hitachi Limited, Tokyo, Japan). Casein solution (1 mL) was added to each centrifuge tube, then different volumes of curcumin solutions were added to obtain curcumin concentrations of 0 × 10^−6^, 2 × 10^−6^, 4 × 10^−6^, 6 × 10^−6^, 8 × 10^−6^, and 10 × 10^−6^ mol/L. Tris-HCl buffer was added to set the volume to 10 mL. The tubes were oscillated to mix evenly. Three sample groups were prepared and incubated in a thermostatic water bath at 298, 305 and 312 K for 30 min. The excitation wavelength was 280 nm, and the excitation spectra of the samples were scanned with an emission wavelength in the range of 300–450 nm.

Fluorescence quenching of casein by curcumin and binding parameters of curcumin to casein can be calculated from the results of fluorescence spectroscopy. The quenching rate constants and quenching constants were analyzed using the Stern–Volmer equation (Equation (1)) [29]:

(1) $$\frac{F_{0}}{F}=1+K_{sv}\left[Q\right]=1+K_{q}\tau_{0}\left[Q\right]$$

where *F* and *F*_0_ are endogenous fluorescence intensities of casein with and without curcumin; *K_q_* is the quenching rate constant; *K_sv_* is the quenching constant; [*Q*] is the curcumin concentration; and *τ*_0_ is the biomolecular fluorescence life time in the absence of quencher being equal to 10^−8^ s.

The binding sites and binding constants of curcumin to casein were analyzed by using Equation (2) [30]:

(2) $$log\left(\frac{F_{0}-F}{F}\right)=logK_{b}+nlog\left[Q\right]$$

where *F*_0_, *F*, and [*Q*] are the same as the Stern−Volmer equation; *K_b_* is the binding constant, and *n* is the number of binding sites.

Thermodynamic parameters were calculated using the thermodynamics equation [31] (Equations (3) and (4)):

(3) $$lnK_{b}=-\frac{\Delta H}{RT}+\frac{\Delta S}{R}$$

(4) ΔG=ΔH−TΔS

where *K_b_* is the binding constant, *T* is the experimental temperature, Δ*H* is the change in enthalpy, Δ*G* is the change in free energy, Δ*S* is entropy change and *R* is the gas constant (8.314 J/(mol·K)).

#### 3.2.3. Ultraviolet-Visible Spectroscopy

Measurements were carried out at 298 K by using ultraviolet-visible spectrophotometer (Beijing General Analytical Instrument Limited Company, TU-1950, Beijing, China). Casein solution (1 mL) was added to each centrifuge tube, then different volumes of curcumin solutions were added to obtain curcumin concentrations of 0 × 10^−6^, 2 × 10^−6^, 4 × 10^−6^, 6 × 10^−6^, 8 × 10^−6^, and 10 × 10^−6^ mol/L. Tris-HCl buffer was added to set the volume to 10 mL. The tubes were oscillated to mix evenly. The samples were placed at 298 K for 20 min. The samples were scanned from 190 to 450 nm.

#### 3.2.4. β-Casein Homology Modeling

As the three-dimensional crystallization structure of β-casein had not been obtained, homology modeling was used to construct its three-dimensional structure. First, the UniProt database (https://www.uniprot.org/ (accessed on 12 March 2021)) was used to search for the sequence information of β-casein (P09116). After local alignment, a search was performed at the RCSB protein database. After comparison, the structures with PDB ID of 5TC1, 2Q2F, and 6O35 were selected as mixed templates. Then, MODELLER (v.9.22) was used for modeling to obtain the optimal three-dimensional conformation. The PROCHECK [32,33] program (https://servicesn.mbi.ucla.edu/PROCHECK/ (accessed on 16 March 2021)) was used to validate and evaluate the three-dimensional protein model.

#### 3.2.5. Molecular Docking

The preliminary three-dimensional structure of curcumin was obtained from Pubchem Compound (https://pubchem.ncbi.nlm.nih.gov (accessed on 17 March 2021)) in NCBI (Pubchem CID: 969516). The structure of β-casein was obtained from the aforementioned homology modeling. YASARA [34] (v.20.10.4) was used for energy minimization to obtain preliminary optimized structures. The setting of different pH values (pH 7.4 and 2.0) was achieved by simulating the different protonation states of amino acid residues under the corresponding pH conditions. The β-casein-curcumin complex was used as the center and expanded 5.0 Å in all directions to obtain a cube. Curcumin and β-casein were considered flexible and rigid during the 100docking runs process. The 100-docking runs process performed in triplicate under each pH condition. Furthermore, the conformation with the lowest energy was selected as the docking result. PyMol (v.2.4.0a0) and LigPlot (v.2.2) was used to plot stable three-dimensional and two-dimensional images with the lowest energy and analyzed the interactions between curcumin and β-casein.

#### 3.2.6. MD Simulation

YASARA was used for MD simulation and Visual Molecular Dynamics [35] (v.1.9.3) was used for visualization analysis. AutoSMILES utility [36] involved in YASARA optimized the structure of the curcumin, generated a topology file and a structure file, and assigned AM1BCC charge to the atom in the topology file. The bond types were assigned by the general AMBER force field. The AMBER14 force field was used for β-casein. Before the simulation, periodic boundary conditions were employed. The lowest energy conformations obtained by three replicates of docking experiments were used as the initial conformations for MD simulation. Firstly, the corresponding pH and temperature (298 K and pH 7.4, 333 K and pH 7.4, 298 K and pH 2.0, and 333 K and pH 2.0) was set. Secondly, the docked β-casein-curcumin complex was used as the center, and an expansion of 10 Å in all directions was performed to construct a cube. Then, TIP3P water molecules were added to fill before sodium ions (Na^+^) or chloride ions (Cl^−^) ions was added to neutralize the system charge. Finally, after energy minimization of the system, 60 ns MD simulation was performed. The MD simulation was carried out with a 2 fs time step, and energy and trajectory coordinates were recorded every 20 ps. After the 60 ns simulation was completed, the md_analyze tool in YASARA was used to analyze changes in root mean square deviation (RMSD), hydrogen bond, system energy and secondary structure with time.

#### 3.2.7. Weak Interaction Analysis

The independent gradient model [37] was used to study the interactions between curcumin and β-casein. Based on the MD simulation results, one steady-state frame in the last 10 ns was selected for weak interaction analysis. The Multiwfn (v.3.7) program [38] was used to calculate the independent gradient model before Visual Molecular Dynamics was used for visualization analysis.

#### 3.2.8. Statistical Analysis

All experiments were performed in triplicate. Data were shown as mean ± standard deviation. The significance of the difference between the measured means was assessed using one-way analysis of variance with Turkey test at the 0.05 probability level in the SPSS software (version 14.0 demo; SPSS Inc., Chicago, IL, USA).

## 4. Conclusions

The interactions between curcumin and β-casein at different temperatures and pH values were illustrated by employing fluorescence, ultraviolet-visible spectroscopy, and MD simulation. The *K_q_* values under different conditions were greater than the maximum diffusion collision quenching constant (2.0 × 10^10^ L/(mol·s)), showing that the intrinsic fluorescence of casein was quenched by curcumin through static quenching mechanism. The binding of curcumin to casein was mainly driven by electrostatic interaction, as evidenced by negative values of Δ*H* and positive values of Δ*S*. The binding energy of β-casein-curcumin complex (−131.07 kJ/mol) was lower at pH 2.0 and 298 K among four conditions, indicating that β-casein-curcumin complex was relatively stable at pH 2.0 and 298 K. This study preliminarily elucidated the interactions between curcumin and β-casein, which could provide a theoretical basis for expanding the application of curcumin-rich casein foods in the food industry.

## Figures and Tables

**Table 1:** Binding and thermodynamic parameters of casein-curcumin complex at different pH values and temperatures.

pH	*T*/K	*K_sv_* × 10^4^/(L/mol)	*K_q_* × 10^12^/(L/(mol·s))	*n*	*K_b_* × 10^4^/(L/mol)	Δ*H*/(kJ/mol)	Δ*S*/(J/(mol·K))	Δ*G*/(kJ/mol)
7.4	298	3.67 ± 0.19	3.67 ± 0.19	1.59 ± 0.14	3.98 ± 0.26	−8.43 ± 0.10	59.93 ± 0.28	−17.87 ± 0.09
	305	3.49 ± 0.07	3.49 ± 0.07	1.35 ± 0.08	3.90 ± 0.15	−8.43 ± 0.10	59.93 ± 0.28	−18.29 ± 0.10
	312	3.42 ± 0.07	3.42 ± 0.07	1.01 ± 0.01	3.41 ± 0.06	−8.43 ± 0.10	59.93 ± 0.28	−18.71 ± 0.09
2.0	298	6.28 ± 0.05	6.28 ± 0.05	1.13 ± 0.02	6.48 ± 0.03	−6.84 ± 0.11	69.21 ± 0.40	−20.63 ± 0.12
	305	5.98 ± 0.09	5.98 ± 0.09	1.17 ± 0.05	6.17 ± 0.05	−6.84 ± 0.11	69.21 ± 0.40	−21.12 ± 0.12
	312	5.74 ± 0.09	5.74 ± 0.09	1.07 ± 0.07	5.73 ± 0.03	−6.84 ± 0.11	69.21 ± 0.40	−21.60 ± 0.12

**Table 2:** Binding energy and dissociation conformation of all conformations obtained by docking of curcumin to β-casein.

pH	Conformation	*E_b_*/(kcal/mol)	*K_d_*/(× 10^−7^ mol/L)	Present Interacting Receptor Residues
7.4	1	−7.01	0.73	10
	2	−6.42	1.90	11
	3	−6.38	2.10	10
	4	−6.34	2.20	11
	5	−6.15	3.10	6
	6	−6.08	3.50	7
	7	−5.67	7.10	10
	8	−5.58	8.20	9
	9	−5.54	8.70	9
2.0	1	−7.53	0.30	11
	2	−7.17	0.55	9
	3	−6.79	1.00	10
	4	−6.62	1.40	12
	5	−6.10	3.40	7
	6	−5.94	4.50	8
	7	−5.89	4.90	8
	8	−5.72	6.40	9

**Table 3:** Various energy items in the system.

pH	*T*/K	Δ*E_vdw_*/ (kJ/mol)	Δ*E_elec_*/ (kJ/mol)	Δ*E_inter_*/(kJ/mol)				Δ*E*/ (kJ/mol)	*E_bind_*/ (kJ/mol)
				Δ*E_bond_*	Δ*E_angle_*	Δ*E_dihedral_*	Δ*E_planarity_*		
7.4	298	2.14 × 10^5^	−1.61 × 10^6^	1.04 × 10^5^	5.69 × 10^4^	5.22 × 10^4^	397.52	−1.18 × 10^6^	−88.85
	333	2.33 × 10^5^	−1.78 × 10^6^	1.29 × 10^5^	6.97 × 10^4^	5.29 × 10^4^	440.38	−1.30 × 10^6^	−35.70
2.0	298	3.36 × 10^5^	−2.44 × 10^6^	1.59 × 10^5^	8.40 × 10^4^	5.08 × 10^4^	398.18	−1.81 × 10^6^	−131.07
	333	2.58 × 10^5^	−1.95 × 10^6^	1.42 × 10^5^	7.63 × 10^4^	5.11 × 10^4^	445.60	−1.42 × 10^6^	−79.28

**Table 4:** Effect of temperature and pH on the composition of secondary structure of β-casein and β-casein-curcumin complex.

	pH	*T*/K	α-Helix Content/%	β-Sheet Content/%	Turn Content/%	Random Coil Content/%
β-Casein	7.4	298	21.47	4.10	20.11	50.72
		333	21.93	5.57	21.56	47.18
	2.0	298	26.27	1.63	17.75	51.89
		333	27.29	1.83	21.74	46.81
Complex	7.4	298	15.56	3.71	20.24	55.50
		333	19.22	5.90	20.73	50.36
	2.0	298	19.69	0.84	18.64	57.16
		333	24.18	2.63	21.05	47.87

## Data Availability

Not applicable.

## Sample Availability

Not available.
